# Ulnar Nerve Granuloma Presenting as a Peripheral Nerve Sheath Tumor: A Case Report and Literature Review

**DOI:** 10.1055/a-2667-7286

**Published:** 2025-08-19

**Authors:** John K. Yue, Jia-Shu Chen, Mahmoud M. Elguindy, Vivian Tang, Ryan Tripathy, Allison R. Bond, Alexander A. Aabedi, Vinil N. Shah, Arie Perry, Andrew W. Bollen, Dong Heun Lee, Line G. Jacques

**Affiliations:** 1 Department of Neurological Surgery, University of California, San Francisco, San Francisco, California, United States; 2 Department of Pathology, University of California, San Francisco, San Francisco, California, United States; 3 Department of Neurological Surgery, University of California, Los Angeles, Los Angeles, California, United States; 4 Division of Infectious Diseases, Department of Medicine, University of California, San Francisco, San Francisco, California, United States; 5 Department of Radiology and Biomedical Imaging, University of California, San Francisco, San Francisco, California, United States

**Keywords:** foreign body reaction, granuloma, infection, peripheral nerve sheath tumor, ulnar nerve

## Abstract

Peripheral nerve masses have a wide differential diagnosis; however, there is no established diagnostic framework for evaluating non-neoplastic etiologies, such as inflammatory or infectious lesions. Here, we present a rare case of an ulnar nerve granuloma that initially mimicked a peripheral nerve sheath tumor (PNST) on imaging and clinical presentation to elucidate the relevant medical history, imaging, and histology that aid in distinguishing inflammatory, infectious, and neoplastic peripheral nerve lesions. An 85-year-old man with melanoma and multiple prior right elbow surgeries presented with right-hand weakness and a rapidly enlarging gadolinium-enhancing ulnar nerve mass suggestive of a PNST that warranted surgical resection. Surgical histology showed a necrotizing granulomatous lesion that then became most concerning for a parasitic infection. However, broad serum and histologic testing by the Centers for Disease Control and Prevention were all ultimately negative. The final diagnosis was an inflammatory reaction to a retained foreign body from his prior elbow surgeries. In summary, surgery and comprehensive histologic workup are required for diagnosing granulomatous peripheral nerve lesions that mimic PNSTs on imaging and infection on histology.

## Introduction

Soft tissue masses associated with the peripheral nervous system encompass numerous etiologies, including neoplastic, infectious, inflammatory, vascular, traumatic, and other categories. Peripheral nerve sheath tumors (PNSTs) account for approximately 20% of soft tissue masses, and MRI has revealed distinctive features of these neurogenic tumors. However, most soft tissue PNSTs show non-specific MRI features, 1 thereby other soft tissue and/or peripheral nerve pathologies can mimic the appearance of a PNST on MRI. Inflammatory responses to trauma, foreign bodies, and infectious organisms represent the next most common etiology that present as a peripheral nerve mass and cannot be excluded until diagnostic tissue has been obtained. For example, traumatic neuromas will exhibit proximal continuity with the nerve, which appears consistent with the “tail sign” commonly found in PNSTs, whereas infectious masses traditionally have rim enhancement like malignant PNSTs. Herein, we report the case of an ulnar mass that initially appeared consistent with a PNST on imaging, was then discovered to be a granulomatous mass on histology suspicious for parasitic infection after surgical resection, but ultimately determined to be an inflammatory foreign body reaction after comprehensive pathological assessment (i.e., “textiloma” or “gossypiboma”). This presentation necessitated a unique and infrequently performed diagnostic evaluation of infectious and inflammatory peripheral nerve masses to identify the etiology. A comprehensive literature review and differential diagnosis for peripheral nerve masses is provided with corresponding distinctive imaging and pathological findings to navigate similar diagnostic dilemmas in future presentations ( Table 1 ).

**Table 1:** Differential diagnosis for mass lesions involving peripheral nerves

Lesion type	History and physical exam	MRI characteristics	Histopathologic characteristics
Benign peripheral nerve sheath tumors			
Intraneural perineurioma 5 21	- Rare, slow-growing mass - Mononeuropathy	- Thickened nerve segment - Fusiform shape - T1 hypointensity - T2 hyperintensity - Homogenous contrast enhancement	- “Onion bulb” appearance - Perineural cells - +EMA - +S-100
Neurofibroma 5 21	- Slow-growing mass - Found in subcutaneous tissue or peripheral nerves - Associated with NF-1 or NF-2 - Asymptomatic unless significant mass effect	- Atrophy of associated muscles - T1 iso-to hyperintense - T2 hyperintense ± central hypointensity (“target sign”)	- Unencapsulated lesion - Often intraneural - Matrix of Schwann cells, fibroblasts, mast cells, and myelinated and unmyelinated axons
Schwannoma 5 21	- Slow-growing mass - Found in subcutaneous tissue or peripheral nerves - Associated with NF-1 or NF-2 - Asymptomatic unless significant mass effect	- Atrophy of associated muscles - T1 iso- to hyperintense - T2 hyperintense ± central hypointensity (“target sign”)	- Encapsulated tumor - Pure Schwann cell proliferation - Highly ordered cellular and loose myxoid components - +S-100
Benign peripheral non-nerve sheath tumors			
Desmoid-type fibromatosis 5 22 23	- Firm mass - Can involve peripheral nerves - Neuropathic symptoms attributed to mass effect	- Fascicular enlargement of peripheral nerve - T1 hypo- to isointense - T2 hypo- to isointense	- Local infiltration of fibroblasts in a collagen matrix
Hemangioblastoma 5 24 25	- Rare mass - Associated with VHL syndrome - Progressive neurological symptoms	- Prominent vessels - T1 hypo-to iso-intense, - T2 iso-to hyper-intense - Contrast enhancement	- Well-circumscribed lesion - Microvacuolated tumor cells - Low mitotic activity - Rich capillary network - +Inhibin - ±NSE - ±S100
Morton's neuroma 21	- Mass of the plantar digital nerve - Typically in middle-aged women - Associated with foot numbness and radiating pain exacerbated by lateral compression	- T1 hypointense - T2 hypointense	- Degeneration and fibrosis of epineural and perineural tissue - Inflammatory reaction of surrounding soft tissues
Neural fibrolipoma 21	- Slow-growing mass - Typically found at birth affecting the median or ulnar nerve - Associated with macrodactyly - Symptoms with mass effect	- Enlargement of affected nerve with cable-like appearance - T1 hyperintense - T2 hypo- or isointense	- Adipose and fibrous tissue infiltration of nerve
Nerve sheath ganglion 5 21 26	- Rare tender mass - Typically associated with peroneal nerve - Swelling and neuropathic pain and/or weakness due to mass effect	- Cystic mass closely associated with affected nerve - ±T2 hyperintensity of nerve - Contrast enhancement of cyst walls	- Cystic structures in epineurium - Clear jelly-like fluid grossly
Malignant peripheral nerve tumors			
Lymphoma 21 27	- Symptoms associated with infiltration of peripheral nerve by primary lymphoma - May also affect peripheral nerve by mass effect	- Uniform thickening - T2 iso- to hyperintense	- Enlarged atypical lymphocytes - Frequent mitoses - Usually B-cell lineage
Malignant peripheral nerve sheath 1 26	- Rapidly enlarging mass - Typically presenting in the proximal peripheral nerves of the upper and lower extremities - Often associated with pain and neurological symptoms - Half of cases arise from NF-1 associated neurofibroma	- Similar to BPNT but may be larger with more lobulation, heterogeneity - Peripheral contrast enhancement	- Fascicular pattern of spindle cells - Wavy or comma-shaped nuclei - High mitotic count - Necrosis - ±Focal S100
Metastatic carcinoma 21	- Typically originating from breast, lung, or melanoma primary cancer	- Variable - Consistent with tissue of origin	- Large atypical epithelioid cells - Frequent mitoses - Necrosis - Immunologic profile similar to primary

Abbrevirations: EMA, epithelial membrane antigen; NF, neurofibromatosis; NSE, neuron-specific enolase; S-100, calcium-binding protein.

## Case Presentation

**Fig. 1:**
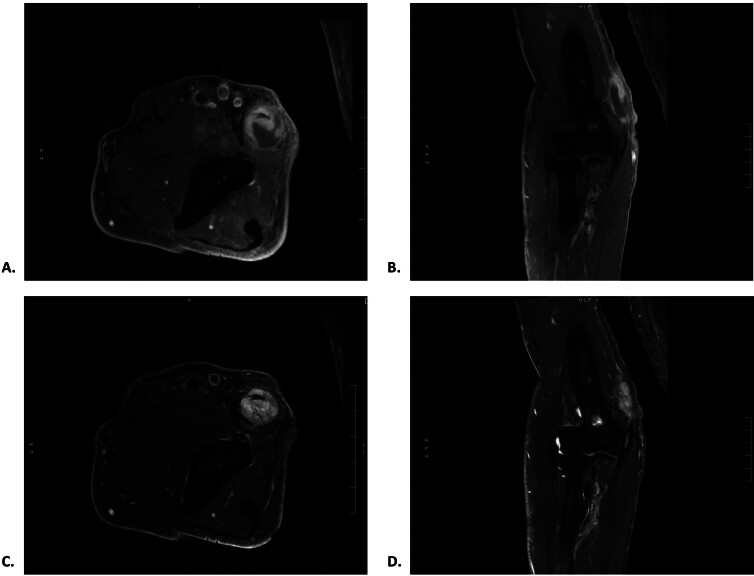
Preoperative magnetic resonance neurogram of the patient's right upper extremity demonstrating a homogenously enhancing, T2 sequence hyperintense, centrally necrotic, well-circumscribed, 1.7 cm (anteroposterior) × 1.5 cm (transverse) × 3.6 cm (craniocaudal) mass in continuity with the ulnar nerve. ( **A** ) Axial T1 postcontrast sequence. ( **B** ) Coronal T1 postcontrast sequence. ( **C** ) Axial T2 sequence. ( **D** ) Coronal T2 sequence.

An 85-year-old male with a history of melanoma, basal cell carcinoma, frequent international travel, and agricultural occupation, and right ulnar nerve transposition 10 years ago for a traumatic, occupational elbow injury, presented for evaluation of a rapidly enlarging right elbow mass associated with radicular pain and paresthesia. One year prior, he had noted a different painful, enlarging mass in his right elbow that was resected 4 months earlier at an outside institution and pathologically diagnosed as a lipoma. The lipoma resection was complicated by a surgical site seroma that developed a week after the surgery and was subsequently drained by his primary care provider. However, the mass returned, prompting an MRI with contrast of his right upper extremity that demonstrated a 1.7 cm × 1.5 cm × 3.6 cm T2-hyperintense lesion with central necrosis and homogenous, peripheral gadolinium-enhancement along the ulnar nerve suspicious for a malignant PNST ( Fig. 1 ), which prompted referral to our clinic. On examination, a soft, non-mobile, and non-tender mass was noted over the right medial epicondyle. The patient had decreased sensation in the right fourth and fifth digits with associated dysesthesias and weakness (strength 3/5: fourth and fifth lumbricals, 3/5: digitus impudicus, 2/5: opponens digiti minimi, 3/5: flexor digitorum profundus). He also had significant muscular atrophy of the right dorsal interossei and hypothenar muscles and difficulty making a fist.

**Fig. 2:**
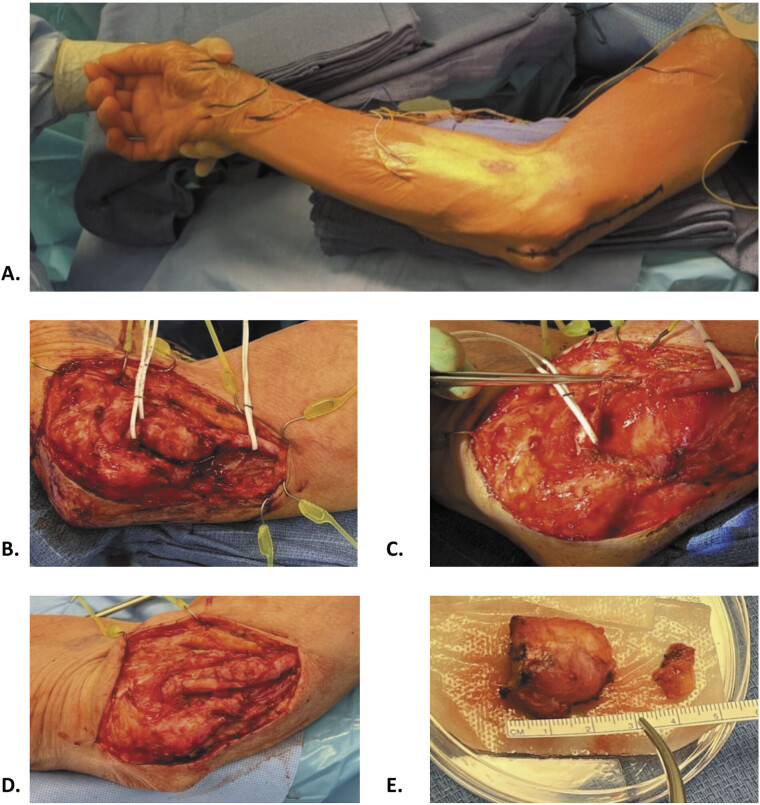
Intraoperative photos demonstrating: ( **A** ) Supine positioning, with the right upper extremity externally rotated and flexed to 90 degrees. ( **B** ) Ulnar nerve mass exposed in situ. ( **C** ) Ulnar nerve in discontinuity following neurolysis and resection of the mass at the level of the medial epicondyle. ( **D** ) Ulnar nerve following repair with allograft interposition and distal nerve transfer from the anterior interosseous nerve at the pronator quadratus. ( **E** ) Ulnar nerve mass following en bloc resection.

**Fig. 3:**
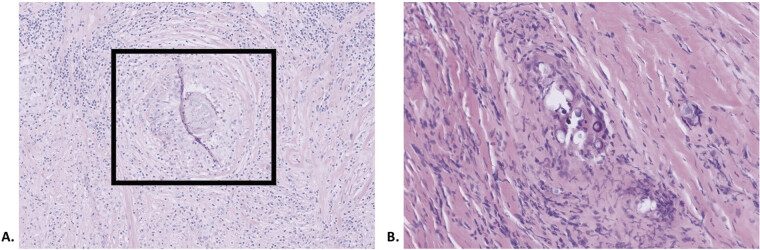
Pathology sections with hematoxylin and eosin staining of the ulnar nerve mass demonstrating: ( **A** ) Extensive granulomatous inflammation with central necrosis around a prominent organism versus foreign body. ( **B** ) Multinucleated giant cells containing calcified and degenerated cystic structures suggestive of refractile capsules.

The patient underwent resection of the mass given its rapidly progressive nature, unclear diagnosis, and associated neurological deficits. Intraoperatively, the mass was hard, vascularized, and inseparable from the ulnar nerve due to dense infiltration ( Fig. 2 ). The mass was resected from the nerve en bloc by first exposing the nerve in its entirety via motor evoked potential and somatosensory evoked potential neuromonitoring. The ulnar nerve gap was repaired via allograft interposition. Distal nerve transfer from the anterior interosseous nerve at the pronator quadratus was performed to provide the best chance for reinnervation and regaining intrinsic hand muscle function. Postoperatively, pathology demonstrated necrotizing granulomatous inflammation around a central structure, focally showing multinucleated giant cells with calcified, cystic structures and refractile capsules, most suggestive of a parasite versus central foreign body ( Fig. 3 ). Stains for fungi, acid-fast bacilli, and spirochetes were all negative. Comprehensive infectious diagnostic tests, including bacterial and fungal cultures, universal polymerase chain reaction, and parasitic tests for leprosy, *Mycobacterium* , *Leishmania* , *Strongyloides* , *Echinococcus* , *Cysticercus* , and *Toxoplasma* , were also negative.

Given the complex differential and the public health importance of recognizing and reporting a potentially indolent parasitic infection, the Parasite Division of the Centers for Disease Control and Prevention conducted an evaluation at the request of our institution's Infectious Disease service. Based on the negative infectious workup and granulomatous, inflammatory pathology associated with a suspicious central structure, the mass was ultimately diagnosed as a foreign body reaction likely secondary to retained material from the patient's prior elbow operations.

Postoperatively, the patient underwent physical therapy and continues to follow up with our neurosurgery and infectious disease teams. At 1-year follow-up, the patient endorsed intermittent tingling along the right ulnar distribution but improved motor strength and muscle bulk of intrinsic hand muscles, and no recurrence of the granulomatous mass.

## Discussion

Extra- and intraneural masses with peripheral nerve involvement encompass a broad differential, a summary of which is presented in Table 1 via literature review. Clinical characteristics and MRI features help narrow the differential; however, pathology is required for a conclusive diagnosis.

Benign PNSTs, such as schwannomas and neurofibromas, are the most common etiology, comprising upwards of 90% of all PNSTs. 2 On T1 postcontrast, they have central enhancement and peripheral hypointensity due to their central fibrocollagenous core and surrounding myxomatous tissue. Furthermore, on T2 imaging, there is commonly heterogenous hyperintensity with central hypointensity, creating the commonly affiliated “target sign.” 3 The patient's imaging did not exhibit these traditional patterns as seen in his peripherally enhancing T1 and homogeneously hyperintense T2 studies ( Fig. 1 ). However, benign peripheral nerve sheath tumors (BPNSTs) remained on the differential given its epidemiology. Conversely, the patient's clinical picture and imaging appeared more consistent with a malignant PNST. Concern for malignant peripheral nerve sheath tumors (MPNSTs) increases in individuals with neurofibromatosis type-1, prior irradiation, and/or symptomatic masses that demonstrate rapid growth, as seen here. 1 4 On imaging, MPNSTs exhibit peripheral enhancement as opposed to central enhancement because of their cystic, necrotic cores. While the prevalence of MPNSTs is lower, the patient's clinical and imaging features kept MPNSTs reasonably high on the differential. However, multiple pathologies have similar MRI characteristics with BPNSTs and MPNSTs, including nerve sheath ganglions, infection, and foreign body reactions, all of which are distinguished on pathology. Nerve sheath ganglions are cystic and gelatinous, while infection and foreign body reactions are characterized by granulomatous histology. 5 Thus, surgery was warranted not only for treatment of worsening neurological deficits but also for diagnosis.

On microscopic examination, the mass did not suggest either a BPNST or MPNST. Pathology showed granulomatous inflammation with multinucleated giant cells and central necrosis surrounding a structure suspicious for a metazoan parasite on H&E. Overall, histopathology suggested infectious, autoimmune, or foreign tissue reaction as the most likely underlying etiology ( Fig. 3 ). With regards to peripheral nerve infections, many viral, bacterial, and parasitic pathogens can lead to peripheral neuropathy, most commonly *Borrelia burgdorferi* , *Corynebacterium diphtheriae* , varicella zoster virus, human immunodeficiency virus, *Giardia* , and *Entamoeba histolytica* . 6 7 These, however, do not present as masses of the nerve itself. Other parasites cause deficits from local mass effect, including *Echinococcus granulosus* , *Taenia solium* , *Loa loa* , *Leishmania* , *Schistosoma* , and *Strongyloides stercoralis* . 8 9 10 From an infectious standpoint, though, the most common cause of peripheral nerve masses is *Mycobacterium leprae* . 11 The tuberculoid form of *M. leprae* presents as a cold intraneural abscess with calcifications or granuloma, most commonly of the ulnar nerve, 12 with multinucleated giant cells from a cell-mediated immune response. 13 Additionally, on MRI, the lesion appears T2-hyperintense with peripheral enhancement and central necrosis. 12 14 15 This constellation of findings, in conjunction with the patient's frequent travel to Mexico and South Africa and agricultural occupation, raised genuine concern for leprosy. However, leprosy lesions are anesthetic, and histology may show enlarged peripheral nerves with acid-fast bacilli, none of which were present. 6 While pure neural leprosy without skin findings can develop, it is rare and manifests in approximately 10% of patients. 14 Ultimately, the patient's infectious workup was all negative, making infection unlikely.

Given a negative infectious workup and the granulomatous appearance, the consensus conclusion was that the mass represented a foreign body reaction, likely secondary to the patient's multiple procedures in that area. The timeline for foreign body reactions becoming symptomatic has significant variance, spanning months to years from the initial surgery, which can make establishing the diagnosis difficult when the incident was long ago and not formally elicited in the history. There have been some reported cases of foreign body peripheral nerve granulomas mistaken for MPNSTs, 16 17 and vice versa, due to overlapping, characteristic imaging findings. Foreign body reactions tend to have peripheral contrast enhancement from ongoing angiogenesis and collagen growth encapsulating the foreign body, T2 hyperintensity, and perilesional edema in cases of abscess, which can be mistaken for MPNSTs in the setting of rapidly progressing lesions. An important distinction between foreign body reactions, BPNSTs, and MPNSTs, however, is on T1 imaging, where foreign body granulomas have greater hypointensity and the latter two are more hyperintense, with MPNSTs exhibiting greater heterogeneity. 18 It is important to note that MRI lack the sensitivity and specificity to routinely identify foreign bodies and can lead to misdiagnosis in the absence of further workup. No obvious retained surgical item was identified grossly or microscopically, which is consistent with the majority of reported histopathology. 19 Another etiology to consider is foreign body reaction to hemostatic material intentionally left in the surgical cavity. Multiple cases of inflammatory reactions to oxidized cellulose that mimic abscesses but yield no organism growth have been identified days to weeks postoperatively. 20 We do not know whether oxidized cellulose was utilized in the patient's previous operations, but it is another etiology to consider.

## Conclusion

In conclusion, this is the first report of an inflammatory foreign body reaction leading to a granulomatous peripheral nerve mass that was originally suspected to be due to an infectious organism because of its histopathology. There is considerable overlap in the imaging of peripheral nerve masses, making a detailed history, pathological tissue evaluation, and laboratory testing necessary. While granulomatous peripheral nerve masses should raise concern for an infectious process and trigger a comprehensive workup, the differential should remain broad, as it is not uniquely specific for an infection. In patients with a history of peripheral nerve surgery and/or trauma, common postoperative complications and their involvement of the peripheral nerve, as discussed here, should be considered at workup for subsequent peripheral nerve deficits.
